# Metalloproteinases in Ovarian Cancer

**DOI:** 10.3390/ijms22073403

**Published:** 2021-03-26

**Authors:** Preston Carey, Ethan Low, Elizabeth Harper, M. Sharon Stack

**Affiliations:** 1 Harper Cancer Research Institute, University of Notre Dame, South Bend, IN 46617, USA; pcarey1@nd.edu (P.C.); elow@nd.edu (E.L.); eharper1@nd.edu (E.H.); 2 Department of Preprofessional Studies, University of Notre Dame, Notre Dame, IN 46556, USA; 3 Department of Chemistry & Biochemistry, University of Notre Dame, Notre Dame, IN 46556, USA; 4 Integrated Biomedical Sciences Graduate Program, University of Notre Dame, Notre Dame, IN 46556, USA

**Keywords:** ovarian cancer, proteolysis, proteases, extracellular matrix, mesothelial cells, peritoneum, mesenchymal, matrix metalloproteinase

## Abstract

Proteases play a crucial role in the progression and metastasis of ovarian cancer. Pericellular protein degradation and fragmentation along with remodeling of the extracellular matrix (ECM) is accomplished by numerous proteases that are present in the ovarian tumor microenvironment. Several proteolytic processes have been linked to cancer progression, particularly those facilitated by the matrix metalloproteinase (MMP) family. These proteases have been linked to enhanced migratory ability, extracellular matrix breakdown, and development of support systems for tumors. Several studies have reported the direct involvement of MMPs with ovarian cancer, as well as their mechanisms of action in the tumor microenvironment. MMPs play a key role in upregulating transcription factors, as well as the breakdown of structural proteins like collagen. Proteolytic mechanisms have been shown to enhance the ability of ovarian cancer cells to migrate and adhere to secondary sites allowing for efficient metastasis. Furthermore, angiogenesis for tumor growth and development of metastatic implants is influenced by upregulation of certain proteases, including MMPs. While proteases are produced normally in vivo, they can be upregulated by cancer-associated mutations, tumor–microenvironment interaction, stress-induced catecholamine production, and age-related pathologies. This review outlines the important role of proteases throughout ovarian cancer progression and metastasis.

## 1. Introduction

Ovarian cancer (OvCa) is the deadliest gynecological cancer, with an overall five year survival rate of only 48.8% [1]. The low survival rate is likely due to a lack of clinical symptoms at early stages of metastasis [1,2]. Therefore, investigations into the method of metastasis in OvCa are warranted in order to find novel therapeutic strategies. Spreading OvCa cells must penetrate the mesothelial cell layer and invade into the sub-mesothelial extracellular matrix (ECM) in order to establish metastatic lesions throughout the peritoneum [3,4]. Both tumorigenesis and metastasis of OvCa are influenced by proteases, a class of enzymes responsible for the catabolism of proteins [5]. Proteases regulate a diversity of biological activities and therefore have a role in DNA transcription, cell proliferation and differentiation, tissue morphogenesis and remodeling, and apoptosis [5,6]. Cysteine, serine, and metallo-proteases are the main subclasses of proteases which contribute to cancer progression and metastasis [5]. These proteases contribute to migration, invasion, ECM remodeling, inflammation and angiogenesis [5]. Often proteases act together in a cascading fashion to promote regulation of these complex biological processes.

Previous studies have demonstrated that progression, invasiveness, and metastatic ability of OvCa are correlated with matrix metalloproteinase (MMP) activity. The MMP family is composed of 25 zinc-dependent enzymes with similar structural and functional domains [7]. The structural domains can be broken down into: (1) a pro-peptide, which must be cleaved for activation of enzyme; (2) a catalytic domain which contains the zinc-ion binding site; (3) a hydrophobic signal peptide; (4) a hemopexin-like C-terminal domain demonstrating substrate specificity; (5) and a proline-dominated hinge region [8]. MMP substrates include several ECM proteins, such as collagen, elastins, gelatins, and caseins [8], and this activity contributes to cancer progression and metastasis [8]. There are six subdivisions of MMPs based on their catalytic activity. (1) collagenases (MMP-1, 8, 13, 14, and 18) which degrade collagen types I, II, and III; (2) gelatinases (MMP-2 and MMP-9) which degrade collagen type IV and gelatin; (3) stromelysins (MMP-3, 7, 10, 11, 26, and 27) which hydrolyze multiple ECM components such as some collagens, elastin, proteoglycans, and glycoproteins; (4) elastases (MMP-12) which degrade elastin; (5) membrane-type MMPs (MMP-14, 15, 16, 17, 24, and 25) which are have a variety of functions, including the activation of MMP-2; 6) other MMPs (MMP-19, 20, 21, 22, 23, and 28) which are not encompassed by any previous classification [8]. MMPs play important roles in developmental and regulatory processes throughout the body, but their role in proliferation, apoptosis, and angiogenesis of is the focus of this review.

MMPs play significant roles in ovarian carcinoma progression and metastasis. There are over 20 MMPs produced by the body fulfilling multiple biological functions, having both unique and overlapping substrate profiles [9,10,11,12]. Initially, MMPs were recognized for their role in late-stage tumor progression, invasion, and metastasis. However, novel evidence has emerged prompting the discussion of the role of MMPs in early tumorigenesis at primary sites [9]. Various unique and cooperative MMPs contribute to ECM degradation, inflammation, migration and epithelial to mesenchymal transition (EMT) [9]. MMPs can also modulate transcription, contributing further to early events in metastatic dissemination [13]. OvCa exhibits a unique metastatic mechanism, as cells are shed from the primary tumor as individual cells or multicellular aggregates and studies have shown MMPs are involved in these early stages of dissemination [4,14,15].

A key enzyme regulating OvCa metastasis is membrane type-1 matrix metalloproteinase (MT1-MMP, also known as MMP-14) [16]. OvCa cells have an increased expression of MT1-MMP as compared to both normal ovarian tissue and benign tumors [16]. This increase is correlated with increased metastasis and poor patient prognosis [16]. In laboratory models, higher expression of MT1-MMP increases OvCa cell invasiveness [16,17]. MT1-MMP, in conjunction with tissue inhibitor of metalloproteinase-2 (TIMP-2), activates pro-MMP-2 [16]. Both MT1-MMP and MMP-2 are modulated by β1 integrin signaling, indicating that cell-matrix contact can influence matrix remodeling [18].

Both MMP-2 and MMP-9 have also been implicated in OvCa metastasis and contribute to enhanced motility and ECM remodeling [19,20,21]. Specifically, MMP-2-catalyzed cleavage of fibronectin (FN) and vitronectin (VN) allows generation of proteolytic fragments with enhanced adhesive properties and may be involved in tumor cell–mesothelial cell adhesion to initiate metastases [22,23]. MMP-9 has substrate specificity for type IV collagen as well as FN [20]. Cleavage of FN by MMP-9 results in the release of active transforming growth factor-β (TGF-β) which further contributes to EMT [24]. TGF-β upregulates transcription factors that induce transcription of MMPs. Other MMPs contribute in supplementary ways to enable effective matrix penetration and metastatic anchoring of OvCa cells [19].

In addition to their impact on early events in dissemination, proteases play a role in late stages of the metastatic process as well. Following mesothelial cell adhesion and retraction of the monolayer to expose the sub-mesothelial matrix, OvCa cells must remodel the tumor microenvironment to provide sufficient space for cytoskeletal spread to enable proliferation [17] and to enhance nutrient availability. Growth factors like vascular endothelial growth factor (VEGF) contribute to angiogenesis required for nutrient supply and support of secondary tumor growth [25,26]. Additionally, both catecholamine release due to chronic stress and increased plasminogen synthesis due to aging have been shown to modulate angiogenesis and thereby support OvCa tumor growth [27,28].

## 2. Tumorigenesis

OvCa carcinomas have been shown to develop from two precursor cells: the ovarian surface epithelium (OSE) and the fallopian tube epithelium (FTE) [29]. Currently the method of tumorigenesis is modeled as a two-pathway system, differentiating between low- and high-grade neoplasms [30,31]. While exact mechanisms by which OSE or FTE precursors acquire transforming mutations have not been elucidated fully, OvCa cells ultimately present with both epithelial (keratin expression) and mesenchymal (vimentin expression) characteristics [29]. Evidence suggests that MMPs play a role in this process.

### 2.1. Epithelial-to-Mesenchymal Transition

One of the most important initial steps in the development of OvCa is the loss of the normal epithelial cell phenotype. Typical normal OSE cells have both epithelial and mesenchymal characteristics, while FTE exhibit an epithelial phenotype [29]. During initial tumorigenesis, OSE or FTE cells acquire more stable mesenchymal characteristics through an epithelial-mesenchymal transition (EMT) [29,32]. Epithelia are comprised of cells that are intimately connected with each other through lateral cell–cell connections including tight junctions, adherens junctions, and desmosomes [33]. These cell–cell interactions maintain epithelial integrity and limit motility. Tight junctions and adherens junctions form a belt between epithelial cells and anchor the cells to each other through intercellular strands and E-cadherin-containing adherens junctions, respectively [33]. Tight junction disassembly is one of the key early events in EMT and results in redistribution of transmembrane proteins between epithelial cells, and plaque scaffolding proteins between cells and the actin cytoskeleton [34,35]. Adhesion between adherens junctions is maintained by Ca^2+^ dependent homodimeric E-cadherin extracellular binding and is linked via β-catenin and other adaptor proteins to cytoskeletal proteins [36,37]. Adherens junction disassembly is a hallmark of EMT and is accompanied by downstream signaling events which cause rearrangement of the cytoskeleton [33,38].

These events are accompanied by acquisition of a more motile phenotype characterized by loss of the cuboidal epithelial shape and conversion to an elongated spindle shape [29]. Indeed, mesenchymal phenotypes are characterized by enhanced migratory capacity, invasiveness, and increased production of ECM components [39,40]. Evidence has shown that the conversion to the mesenchymal type is sufficient to facilitate metastasis of cancerous cells [36]. Accompanying the loss of E-cadherin is the acquisition of mesenchymal proteins such vimentin and N-cadherin [39]. In some tumor models, mesenchymal cells have been shown to migrate in the ECM along pre-defined matrix tracks as well as rearranging matrix into new tracks to create new migratory pathways [41].

The contribution of MMPs to EMT also functions to promote tumorigenesis and invasion [42,43]. While the roles of MMPs in ECM degradation are well-characterized, new research is elucidating additional roles for these proteases. MMP-9 in particular was demonstrated to play a major role in promoting EMT [44]. MMP-9 expression can be regulated by numerous pathways including specificity protein 1 (SP-1) and can function to modulate cell–cell adhesion by catalyzing E-cadherin ectodomain cleavage [44,45,46,47,48]. These results were confirmed with studies demonstrating that knockout of the MMP-9 gene resulted in more localized concentrations of E-cadherin [49]. E-cadherin is a known epithelial marker and high concentrations would suggest an epithelial cell type [50]. E-cadherin ectodomain shedding correlates with MMP-9 activity and is often accompanied by a transition from a static epithelial state to a mobile mesenchymal phenotype [50]. In addition to E-cadherin acquisition, a decrease in mesenchymal markers such as vimentin and Snail were also observed, leading to a functional reversal of EMT through mesenchymal–epithelial transition (MET) [49]. Thus, MMP-9 functions as an inducer of EMT through its proteolytic action on E-cadherin and concomitant loss of other epithelial markers.

### 2.2. Proteolytic Effects on the TGF-β Signaling Pathway

**Figure 1:**
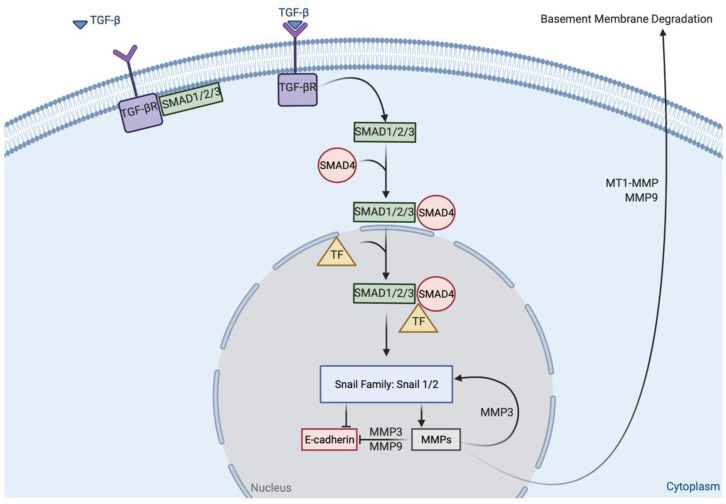
The role of TGF- β in EMT and metastasis. When TGF- β binds to its receptor, SMAD1/2/3 are released into the cytoplasm, where they bind to SMAD4. In the nucleus, the SMAD complex binds to transcription factors (TF) that upregulate the Snail family. Snail1/2 promote MMP production and downregulate E-cadherin. MMP-9 and MMP-3 degrade E-cadherin, reducing the amount of functional E-cadherin available to the cell. MMP-3 also promotes Snail family upregulation in a positive feedback loop. MT1-MMP and MMP-9 degrade the basement membrane, further promoting EMT and metastasis. Figure adapted from [33,51,52].

Transforming growth factor β (TGF-β) has also been shown to play a major role in initiating the EMT conversion early in the tumorigenic process through several mechanisms (Figure 1). Treatment of epithelial cells with TGF-β has been shown to induce a transition from cuboidal to elongated spindle shape concomitant with acquisition of additional mesenchymal characteristics [33]. TGF-β activates Smad1/2/3 signaling that upregulates the Snail family of transcription factors [33]. These factors, in turn, repress expression of epithelial markers such as E-cadherin, desmoplakin, and cytokeratins and induce transcription of several MMPs including MMP-1, MMP-2, MMP-7 and MT1-MMP [51,52,53]. Treatment of cells with broad-spectrum MMP inhibitors in addition to TGF-β mitigated the pro-invasive phenotype caused by TGF-β alone [52]. While promoting MMP transcription, the increased expression of the Snail family of transcription factors was found to have a negative correlation with E-cadherin expression [47,51,53].

Both MMP-3 and MT1-MMP were also shown to influence EMT. MMP-3 was determined to help promote EMT through upregulation of the Snail family transcription factors [21]. MMP-3 was also shown to bind and cleave E-cadherin as a substrate resulting in decreased cell–cell adhesion [54]. Comparatively, MMP-3 and MMP-9 exhibit many of the same effects on expression and EMT. MT1-MMP is expressed in metastatically active OvCa cells [24]. In gastric cancer, MT1-MMP is shown to upregulate EMT proteins including Snail family proteins [55]. Increased expression of MT1-MMP has been shown to increase the formation of MCAs and increase metastatic ability [56]. MCAs have been shown to have higher invasive and adhesive ability relative to individual cells [15].

## 3. Metastasis

Much research has been done on the early metastatic steps in OvCa progression and the proteins that mediate adhesion to the mesothelium and trans-mesothelial migration, which are rate limiting steps in metastasis [23].

### 3.1. Adhesion

The mesothelial monolayer lines the peritoneum and provides a surface for adhesion of OvCa cells during metastasis. These cells contain several proteins to which OvCa cell bind. For example, mucin 16 (Muc16) is a surface-associated mucin that contains a known cancer antigen (Ca125) within its protein sequence and is highly expressed ectopically on OvCa cells [57,58]. This protein promotes tumor cell binding to the mesothelium and provides an initial weak linkage to the mesothelial layer [59,60]. Muc16 preferentially binds mesothelin expressed on peritoneal mesothelial cells [57,59]. After the initial binding of Muc16 to mesothelin, MT1-MMP can catalyze cleavage of Muc16 protein from the extracellular surface [57]. This cleavage may facilitate strong binding between OvCa cells and the mesothelium through integrin-mediated adhesion [57]. MT1-MMP-catalyzed cleavage of Muc16 prior to mesothelial anchorage may also facilitate homotypic association of OvCa cells into multicellular aggregates, providing an additional mechanism for enhanced metastatic propensity [57]. The mesothelial layer is coated in FN and VN, both of which are known substrates of MMP-2 [24,56]. MMP-2 is found at higher concentrations at the leading edge of migratory OvCa cells and regulates ɑvβ3 integrin-mediated adhesion to pericellular FN fragments [61]. Studies show MMP-2 inhibitors decrease the cellular adhesion to the mesothelial surface [23,24,26].

OvCa cells bind to collagen types I -III prevalent in the sub-mesothelial matrix via α2β1 and α3β1 integrins [13,62]. This adhesion and subsequent matrix-induced integrin clustering activates SRC signaling, resulting in increased expression of the transcription factor EGR1. EGR1, in turn, induces MT1-MMP transcription which further modulates mesothelial anchoring, and promotes cleavage and activation of pro-MMP-2 into its active form, resulting in FN degradation [4,13,61,62]. At the point of contact with the host mesothelial cell, proMMP-2 transcription is also upregulated [23].

### 3.2. Invasion

Recent studies have elucidated several important cellular mechanisms involved in tumor–microenvironment interactions at secondary metastatic sites. Once ovarian cancer cells have adhered to the metastatic niche, the anoikis-resistant cells that were present in ascites transition to a proliferative state that can interact with the microenvironment of the omentum and peritoneum, a process facilitated by mesenchymal-to-epithelial transition (MET) [29]. Successful colonization requires intimate interplay between the tumor microenvironment and the metastatic cancer cells.

Ex vivo and xenograft experiments with ovarian cancer cells have demonstrated a microenvironment-induced downregulation of miRNA-193b that drives invasion and proliferation in the omentum [63,64]. This downregulation was found to increase the expression of urokinase (urinary type plasminogen activator, or uPA), a known tumor-associated protease [64]. Plasminogen activation by uPA promotes tumor invasion in lung, breast, and ovarian cancer [19,65,66,67]. In other cancers uPA has been shown to have important roles in angiogenesis as well as migration and ECM remodeling and colocalizes with several MMPs in patient tissues [67,68]. Notably, plasminogen, when cleaved by uPA into plasmin, can degrade many ECM proteins in the basement membrane and activate other protease zymogens [66]. Metastasizing ovarian cancer cells also have the ability to remodel the microenvironment. They can promote metastasis by stimulating peritoneal fibroblasts, mesenchymal stem cells, and tumor-associated macrophages through the expression of the HOXA9 gene [69] and xenograft studies show a correlation between high HOXA9 gene expression and increased ovarian tumor growth [70].

MMPs are integral contributors to ovarian cancer invasion [17,71]. MMP-2 and MMP-9 are found in OvCa patient ascites and aid cancer cell invasion through type IV collagen degradation [72]. MT1-MMP in particular is required for OvCa cell invasion. In addition to activating the pro-MMP-2 zymogen [16], MT1-MMP is an interstitial collagenase that is expressed on the surface of metastasizing OvCa cells and contributes significantly to intra-peritoneal anchoring in the interstitial collagen-rich sub-mesothelial matrix [73]. This is supported by in vitro studies showing that MT1-MMP catalytic domain and phosphorylation site mutations (E240A and T567E, respectively) that disrupt enzymatic activity show significantly reduced collagen invasion relative to cells expressing the wild type enzyme [17,74].

### 3.3. Angiogenesis

Another important process in later stages of metastasis that is regulated by proteolysis is angiogenesis. Angiogenesis, the formation of new blood vessels from pre-existing vasculature, is an established hallmark of cancer [25]. It is an important process for both primary and metastatic tumor growth [25]. Without angiogenesis, a tumor mass is unlikely to exceed the diffusion-limited maximal size of approximately 2 mm^3^ [75]. Various anti-angiogenic therapeutics have been studied and have proven to extend the life of ovarian cancer patients by several months. Most are classified as anti-angiogenic therapeutics because of their inhibition or reduction of vascular endothelial growth factor (VEGF) pathways [76]. VEGF has been shown to stimulate vascular and lymphatic endothelium to form new blood and lymphatic vessels while also regulating the vessel permeability [29]. In the context of normal ovarian physiology, VEGF-induced angiogenesis is essential for normal reproductive function and has been shown to play an important role in the ovulatory cycle [77]. A study using human ovarian carcinomas demonstrated that VEGF-A, VEGF-C, VEGFR-2, and VEGFR-3 were expressed in tumor cells as well as adjacent endothelial cells of blood and lymphatic vessels [78]. The expression of VEGF-C and VEGFR-2 specifically were correlated with increased metastatic ability, including peritoneal metastases outside the pelvic region, lymph node metastases, and positive ascitic cytology [78]. Other in vitro and in vivo experiments have demonstrated that VEGF is involved in both paracrine and autocrine signaling [79,80,81,82].

In addition to their role in adhesion and invasion as previously described, MMPs also play a major role in inducing angiogenesis. MMP-2 downregulation in chicken chorioallantoic membrane models as well as MMP-2 deficiencies in mice are linked to decreased tumor angiogenesis and growth [83,84]. As stated above, MMP-2-catalyzes cleavage of fibronectin (FN) and vitronectin (VN) and facilitates tumor cell–mesothelial cell adhesion that initiates metastases [22,23]. MT1-MMP, an activator of pro-MMP-2 and a potent interstitial collagenase, is also thought to influence cancer cell angiogenesis by promoting the formation of capillary tubes [85]. Similar to MMP-2, MMP-9 cleaves ECM proteins and has substrate specificity for type IV collagen as well as FN [20]. In vivo experiments have confirmed in vitro results that demonstrate the role of MMP-9 and MT1-MMP in angiogenesis, as mice deficient in MMP-9 and MT1-MMP have been shown to have a reduced ability to induce angiogenesis compared to MMP-9 wild type mice [86,87,88].

**Figure 2:**
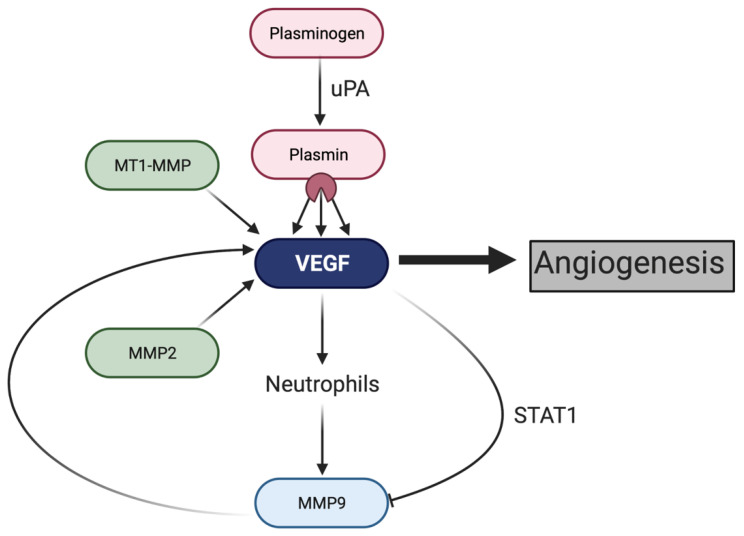
Effects of proteolysis on the VEGF pathway. Plasminogen, when cleaved by uPA into its active form plasmin, cleaves VEGF into different isoforms for multiple biological functions [97]. VEGF recruits neutrophils, which express MMP-9, which encourages VEGF production in a positive feedback loop [92,93]. MT1-MMP and MMP-2 also promote VEGF-mediated angiogenesis [83,87].

The mechanism that underlies MMP-induced angiogenesis is an area of active investigation; however, there is a clear interplay between MMPs and the VEGF pathway (Figure 2) [26]. Specifically, MT1-MMP overexpression can stimulate an increase in VEGF production and angiogenesis in glioblastoma and breast carcinomas models [89,90,91]. MMP-9 as well works to induce angiogenesis by increasing the availability of VEGF in islet cell models [92]. In ovarian cancer cells, VEGF enhances the expression of host MMP-9 in the ovaries due to an increased influx of neutrophils that secrete MMP-9, demonstrating a potential feedback mechanism between VEGF and MMPs [93]. However, in B-cell leukemia cells, it has been shown that VEGF can significantly reduce MMP-9 protein expression in a dose dependent manner, so further research is needed to elucidate the relationship between MMP-9 and VEGF [94]. Although the exact mechanism by which MMPs increase VEGF production requires more research, a study in an adenocarcinoma model suggests that an integrin linked signaling pathway via αvβ3 promotes VEGF-mediated angiogenesis [95,96].

## 4. Host Factors Influencing Proteolysis

**Figure 3:**
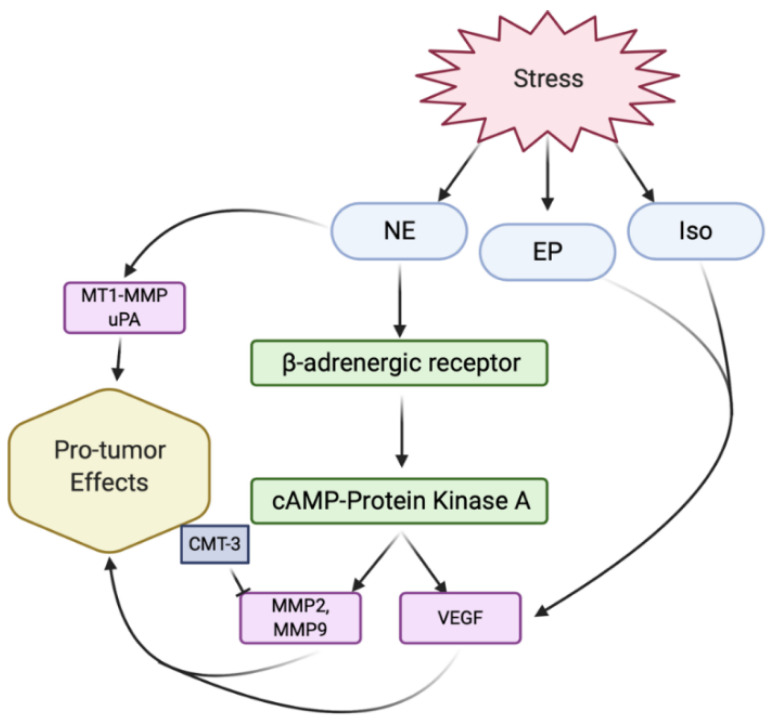
Relationship between stress, proteolysis, and VEGF-mediated angiogenesis. Stress induces expression of norepinephrine (NE), epinephrine (EP), and isoproterenol (Iso) [98]. NE activates β-adrenergic receptors, which activates the cyclic AMP (cAMP)- protein kinase A signaling pathway [27]. This pathway leads to an upregulation of VEGF as well as MMPs, such as MMP-2 and MMP-9 [27]. Other proteases such as MT1-MMP and uPA are upregulated by NE as well, also leading to pro-tumor effects which can be mitigated by CMT-3 [28,99]. EP and Iso have been shown to upregulate VEGF expression as well [98].

Factors such as aging and stress are increased risk factors for many cancers including ovarian cancer. Increased levels of chronic behavioral stress results in higher tissue levels of various catecholamines such as norepinephrine (NE) [98]. In vitro studies demonstrated that NE, epinephrine, and isoproterenol, a β-adrenoceptor agonist, significantly enhanced VEGF production by an endothelial germ cell (EG) ovarian cell line, while NE and isoproterenol significantly enhanced VEGF production by SKOV3 cells [98]. Reverse transcriptase PCR studies demonstrated constitutive expression of β-1 and β-2 adrenergic receptors in both EG and SKOV3 cell lines [98]. The catecholamine-induced increase in VEGF expression was confirmed in mouse models as well [99]. Lastly, human studies of pre-surgical ovarian carcinoma patients have shown that higher NE levels were found in late-stage and higher-grade tumors [100]. In addition, patients with a self-perceived lack of social support were shown to have significantly higher tumor and ascitic NE levels [100]. Human studies of presurgical ovarian carcinoma patients have also found that lower levels of social support were associated with higher serum VEGF levels [101]. These findings demonstrate the potential mechanism for catecholamine-induced increases in VEGF levels in ovarian cancer patients (Figure 3).

In vivo studies have also examined the potential relationship between the increased VEGF levels and increases in proteolytic activity in stress-induced mice. These studies have shown that stress-induced release of catecholamines activates a β-adrenergic receptor that activates a cyclic AMP-protein kinase A signaling pathway on ovarian carcinoma cells to enhance the expression of VEGF, MMP-2 and MMP-9 [27]. Tumors in these stressed animals demonstrate a markedly increased vascularization and a more aggressive growth and spread of malignant cells [27]. Analysis of mRNA expression has also shown that social isolation-induced stress in mice with liver metastases caused an increased expression of MMP-2, MMP-9, MTI-MMP, and uPA in the tumor and liver tissue compared to the control mice [28]. These increases in MMP-2 and MMP-9 have also been confirmed in head, neck, and colon cancers [102,103,104,105].

The enhanced tumor cell invasive potential caused by increased catecholamine release and the subsequent MMP-2 and MMP-9 expression can be successfully abrogated with the pharmacological blockade of MMPs using CMT-3, a broad-spectrum MMP inhibitor, indicating that MMP activity plays an integral role in the pathways of catecholamine-induced increase in tumor cell invasive potential [99]. Ovaries have been shown to have increased levels of sympathetic innervation in ovarian tissue and can upregulate the expression of genes encoding for key enzymes in catecholamine biosynthesis, making this especially relevant for ovarian cancer patients [99,106,107,108,109]. Clinically, MMP-9 secreted by tumor-associated macrophages (TAMs) has been linked to stress [110]. TAMs have been shown to increase tumor progression and therefore play an important role in the relationship between proteolysis and ovarian cancer metastasis [111].

Age has been shown to increase ovarian cancer metastasis in murine models [112]. Aging also significantly upregulates proteases in the microenvironment, suggesting the hypothesis that age-dependent changes in proteolytic activity alter ovarian cancer progression [113]. Interestingly, plasminogen levels are significantly higher in human vitreous of study participants over 50 [114]. As detailed above, plasmin cleaves several relevant proteins in the ovarian tumor microenvironment, such as fibrin and fibronectin, and cancer cell surface molecules such as CDCP1 to facilitate invasion [68,115,116]. Studies on senescent fibroblasts have suggested that increased MMP-3 may be responsible for cell senescence and therefore contribute to age-related pathologies [117].

The role of proteolytic activity on photo-aging has also been studied extensively. Exposure to harmful UV rays has been shown to significantly increase levels of MMPs in skin collagen [118]. These increased levels of MMPs are likely contributors to photoaging or the premature aging of skin as a result of prolonged exposure to ultraviolet radiation [118,119,120]. Although not susceptible to UV exposure, the ovarian cancer microenvironment is rich in interstitial collagens, which are known to acquire altered post-translational modifications with age, so the aging ovarian cancer microenvironment likely has altered susceptibility to MMP-catalyzed proteolytic modification [113].

## 5. Conclusions

**Figure 4:**
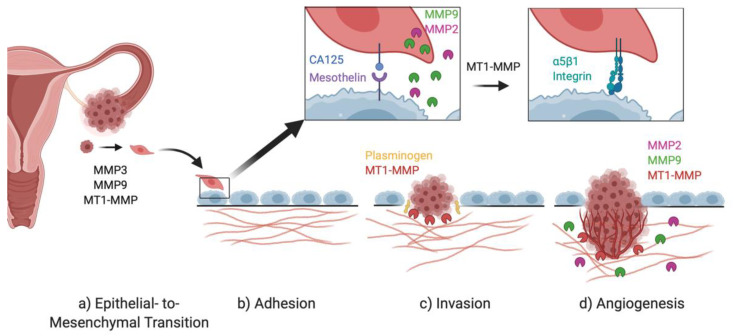
(**a**) Epithelial-to-mesenchymal transition (EMT), mediated by MMP-3, MMP-9, and MT1-MMP, promotes tumor cell detachment from the primary tumor, resulting in diffusion of cancer cells into the peritoneal cavity [44,52], where they (**b**) adhere to the mesothelial monolayer via binding of CA125 on the cancer cell by mesothelin on the mesothelial cell [57]. MMP-9 and MMP-2 are secreted by the cancer cell to cleave fibronectin produced by the mesothelial cell [24]. CA125 is cleaved by MT1-MMP to allow for integrin-mediated adhesion to the fibronectin fragments on the mesothelial cell and to induce mesothelial cell retraction [57]. (**c**) The basement membrane is degraded by MMPs and plasmin and the sub-mesothelial collagen matrix is remodeled by MT1-MMP [72,74]. (**d**) VEGF-mediated angiogenesis is promoted by MT1-MMP, MMP-9, and MMP-2 [83,85,86].

Metalloprotease activity mediates every step of progression in ovarian cancer, from tumorigenesis to metastatic implantation (Figure 4). A key process in OvCa metastasis is EMT, which facilitates initial detachment. Various metalloproteinases are differentially modulated in this process. Most notably, both MMP-9 and MMP-3 can act as inducers of EMT by cleaving E-cadherins and decreasing the expression of epithelial markers [44]. MMPs and Snail transcription factors also function in regulatory transcriptional feedback loops to facilitate events in metastatic progression [21,51].

Adhesion of metastatic cells to the mesothelial layer of the omentum or peritoneum is an additional rate limiting step in metastasis that is regulated by MMPs [57,61]. MT1-MMP contributes to proteolytic remodeling of the mesothelial cell surface while MMP-2 alters pericellular FN and VN deposition to further facilitate adhesion [56,57,61].

Further, the establishment of a metastatic tumor involves the interplay between the tumor microenvironment and malignant cancer cells. In ovarian cancer, the tumor microenvironment can upregulate proteases that influence adhesion, motility, matrix remodeling and angiogenesis [64,65,66,67,68]. Ample evidence supports a role for proteolysis in the expression and processing of VEGF to regulate the angiogenic process [25,26,86,87,88,89,93].

Lastly, additional host factors may control protease-mediated metastatic dissemination. Interesting data link host catecholamine levels, resulting from induced behavioral stress, to enhanced metastasis. [27,28]. Aging and age-related pathologies have also been linked to increased altered matrix structure, protease expression and matrix remodeling capability [114,117,121]. Proteolytic cross talk between the tumor and host microenvironment regulate complex pathobiological processes that contribute to ovarian cancer progression and metastasis.

Extensive studies aimed at targeting MMP activity in a number of cancers ultimately failed in clinical trials due to the broad-spectrum inhibitory profiles of the compounds employed. It can also be reasoned that the late-stage patients enrolled in the trials would not receive the same benefit from MMP inhibition that early-stage patients would, while the potent side effects would not promote patient compliance in the early stages [71]. To this end, researchers have developed more selective MMP inhibitors that have shown some success in the clinic.

Tissue inhibitors of metalloproteinases, or TIMPs, are naturally occurring MMP inhibitors that block activity or activation of various MMPs, and are more specific than most small molecule MMP inhibitors [8]. For example, TIMP-2 has a higher specificity to MMP-2 and TIMP-1 has specificity for MMP-1, MMP-3, and MMP-9 [8]. In ovarian cancer cell lines, TIMP-2 is a potent inhibitor of MT1-MMP and can significantly reduce invasion through inhibiting matrix degradation [122]. In chicken embryo assays, the MMP inhibitor TIMP-1 has been found to decrease angiogenesis [123]. On the other hand, some studies have shown that TIMPs can increase metastatic potential. MT1-MMP, in conjunction with tissue inhibitor of metalloproteinase-2 (TIMP-2), form a complex to activate pro-MMP-2 [16]. Human studies show that high levels of TIMP-1, an MMP-inhibitor that binds preferentially to MMP-9, is associated with reduced overall survival [8,124].

Another way to increase selectivity, and subsequently increase clinical success, is by targeting MMPs with selective monoclonal antibodies (mAb) [125]. Of particular interest in this review is the targeting of MT1-MMP. The first approved mAb was DX-2400, which functions as a competitive inhibitor that selectively inhibits MT1-MMP by blocking substrate binding [125]. It was shown to be effective at reducing invasion, angiogenesis, and tumor growth in a breast cancer model [126,127]. Additionally, there are mAbs designed for MMP-2 and MMP-9 that could prove efficacious for cancer treatment [125].

Given the complexity of the system, targeting metalloprotease activity as a therapeutic modality is not without challenges, and more research is required on the effects of MMP inhibitors. Novel approaches are needed to enhance the specificity of targeted inhibitors and to more precisely modulate their delivery to neoplastic cells.
